# A Capillary-Perfused, Nanocalorimeter Platform for Thermometric Enzyme-Linked Immunosorbent Assay with Femtomole Sensitivity

**DOI:** 10.3390/bios10060071

**Published:** 2020-06-24

**Authors:** Evan Kazura, Ray Mernaugh, Franz Baudenbacher

**Affiliations:** 1 Department of Biomedical Engineering, Vanderbilt University, Nashville, TN 37235, USA; evankazura@gmail.com; 2 Department of Biochemistry, Vanderbilt University School of Medicine, Nashville, TN 37235, USA; mernaurl@gmail.com

**Keywords:** microfabricated calorimeter, ELISA, thermometric ELISA, biosensor, model-assisted signal analysis

## Abstract

Enzyme-catalyzed chemical reactions produce heat. We developed an enclosed, capillary-perfused nanocalorimeter platform for thermometric enzyme-linked immunosorbent assay (TELISA). We used catalase as enzymes to model the thermal characteristics of the micromachined calorimeter. Model-assisted signal analysis was used to calibrate the nanocalorimeter and to determine reagent diffusion, enzyme kinetics, and enzyme concentration. The model-simulated signal closely followed the experimental signal after selecting for the enzyme turnover rate (*kcat*) and the inactivation factor (*InF*), using a known label enzyme amount (*Ea*). Over four discrete runs (*n* = 4), the minimized model root mean square error (RMSE) returned 1.80 ± 0.54 fmol for the 1.5 fmol experiments, and 1.04 ± 0.37 fmol for the 1 fmol experiments. Determination of enzyme parameters through calibration is a necessary step to track changing enzyme kinetic characteristics and improves on previous methods to determine label enzyme amounts on the calorimeter platform. The results obtained using model-system signal analysis for calibration led to significantly improved nanocalorimeter platform performance.

## 1. Introduction

Enzyme-linked immunosorbent assays (ELISAs) are the gold standard for detecting and quantifying antigens and antibodies in biological samples and have been extended to other biological markers for drug discovery and pregnancy tests [1,2]. Most commercially-available ELISA kits utilize enzyme-conjugated reagents (e.g., antibodies) specific for a target analyte to produce a colorimetric, chemiluminescent, or fluorescent signal that can be quantified using a microtiter plate reader [3,4,5]. Although specific and sensitive, many detection methods prevent ELISAs from on-site use [6,7,8]. ELISAs with optical-based detection have been developed for point-of-care (POC) operation, but either lack quantitative results or require samples with particular optical properties [9]. Mattiasson et al. first developed an ELISA with a calorimetric readout of the heat produced by the enzymatic reaction, named thermometric ELISA (TELISA) [10]. TELISA produces a direct readout from the heat produced by the enzymatic reaction. The original TELISA calorimetric biosensor used flow-through columns with immobilized enzymes, requiring large sample volumes greater than a finger prick, ambient temperature controls, and external pumps for liquid handling. Flow-through TELISA achieved a limit of detection (LOD) of 86 picomoles of labeling enzyme for detecting insulin [11]. Although the design of the TELISA successfully demonstrated a proof-of-principle, these factors prevented TELISA from reaching the sensitivity of conventional ELISAs, and the design was not suited for use in POC applications [12,13]. Advances in micromanufacturing have made possible the development of microfluidic calorimeters that can be used as platforms to carry out TELISA. Calorimeters that achieve nanojoule levels of sensitivity are defined as nanocalorimeters. Nanocalorimeter-based TELISAs can rapidly detect small temperature changes in enzyme-based immunoassays to produce fast, quantifiable electronic readouts [14,15]. As such, microfabricated devices used to carry out TELISAs can be competitive with traditional ELISAs. Nestorova et al. showed the feasibility of TELISA without complex temperature control in determining the 8-hydroxy-2-deoxyguansoine (8OHdG) levels in urine [16]. Xu et al. utilized flow-injection analysis TELISA to detect diazepam in beverages, achieving an LOD of 43.8 picomoles with minimal sample pretreatment [17]. Both systems take advantage of TELISA for rapid results without expensive imaging equipment. However, both require external pumps for liquid sample handling, limiting the approach to a lab setting.

Biosensor platforms that can quickly be adapted to new targets are in high demand. Since enzymes used in ELISAs produce heat as a byproduct of an enzyme-catalyzed reaction, a calorimeter becomes an easily adaptable and customizable platform to detect enzymes, as indicators of antigen presence, in TELISAs. Hydrogen peroxide is a commonly used substrate in ELISAs. It is ideal due to its high reaction enthalpy (−98 kJ/mol) and large catalog of reactive enzymes (e.g., catalase or horseradish peroxidase, etc.). Catalase (CAT; Enzyme Commission number (EC) 1.11.1.6) catalyzes the decomposition of H_2_O_2_ into water and oxygen, a well-characterized reaction. CAT also features fast turnover rates, so small amounts of the enzyme can turn over substrate quickly. This maximizes sensitivity when labeling small amounts of target analyte with an enzyme. TELISA relies on knowing the reaction kinetics, which degrade over time and change in different conditions. CAT becomes inactivated after catalyzing 10^7^ hydrogen peroxide molecules, adding a complication to the assay when using high-substrate concentrations over a long duration [18]. Using these enzymes as labels creates adaptable biosensors that apply the same transduction method and simply require reliable labeling of the target analyte. The TELISA approach reads the signal as the reaction is occurring, producing real-time results.

We have successfully developed highly sensitive nanocalorimeter TELISA platforms and showed the detection of clinically relevant levels of herceptin and phenylalanine in serum [19,20]. The devices feature a small footprint, require nanoliter volumes of sample, and are mass produced by standard batch microfabrication techniques that can be commercially produced at a cost of less than $1 USD per device. In our previous studies, modeling using radial, two-dimensional (2D) simulation guided our nanocalorimeter platform design. However, that approach did not apply to the nanocalorimeter’s microfluidic channel geometry, and did not include the kinetics of chemical reactions. Previous work showed a calorimeter thermal time constant of 325 ms, and an energy sensitivity of 1.4 nJ/Hz^1/2^. For comparison purposes, this translates to an LOD of 25 femtomoles (fmol) for an acid–base neutralization reaction [19]. Nanocalorimeter fluid handling was driven by capillary forces, and assay readouts were obtained via direct voltages, showing promise for POC operation. Although the nanocalorimeter platform’s performance has been explored and is well understood, determining the enzyme amount is challenging. Previously, enzyme-based calorimeter assays were simplified using a phenomenological approach, by solely measuring the total amount of substrate consumed or the decay time for the first several seconds of a heat/voltage-generating reaction to determine results. This method did not exploit the full reaction time course and required reactions with high enthalpies and fast kinetics. A more robust technique that considers the entire time course of the enzymatically-catalyzed reaction would reduce run-to-run variability and decrease uncertainty in TELISA results, increasing the limit of detection.

In the present study, we incorporate finite element numerical modeling, simulating both the enzyme reaction and substrate diffusion, as well as the physical characteristics of a capillary-driven POC calorimeter platform. We use a single, comprehensive model to interrogate the Michaelis–Menten-governed enzyme kinetic reaction, to determine the enzyme amount to extract using the entire time course. We demonstrate the use of model-assisted signal analysis to calibrate enzyme kinetics for experimental sets and determine the enzyme amounts correlated to a target substance in a TELISA operation. This complete numerical approach allowed us to reduce the extrapolated LOD to attomole levels of catalase and achieve experimental detection of femtomoles of catalase on our nanocalorimeter platform as a proof of concept for an adaptable TELISA biosensor.

## 2. Materials and Methods

### 2.1. Nanocalorimeter Platform Layout

**Figure 1:**
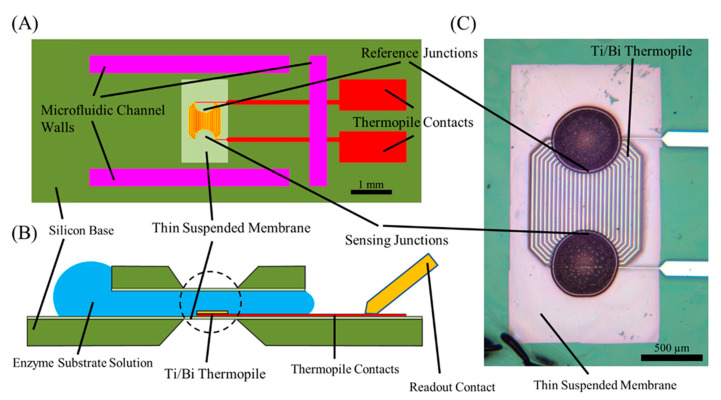
(**A**) Nanocalorimeter platform consisting of an Su-8 polymer thin membrane on a silicon base, Su-8 walls, and a thermopile calorimeter. (**B**) A second Su-8 membrane on silicon seated on the walls forms a microfluidic channel around the calorimeter. The thin membrane thermally isolates the reaction zone, calorimeter sensing, and reference junctions from the environment. Liquid placed at entrance to the microfluidic channel is drawn in by capillary forces, filling the channel without external pumps. (**C**) The calorimeter consists of a 27 junction Bi/Ti thermopile in a differential format. Sensing junctions and reference junctions are each arranged in a semicircle on the freestanding thin membrane. The temperature difference between the sensing junctions and reference junctions generates a proportional voltage differential between the thermopile contacts. Additional details on platform design and construction can be found in Lubbers [21]. Figure adapted from Kazura et al. [19].

Figure 1A shows a top-down representation of the nanocalorimeter platform base. The platform consists of a thermally isolated reaction zone, with a differential thermopile to measure reaction enthalpies. The reaction zone is integrated in a microfluidic channel, which consists of two thin membranes separated by two strips of thick photoresist forming the channel walls. The two membranes are supported by a silicon wafer. A thin layer of the epoxy-based polymer photoresist Su-8 (MicroChem) is hard-baked on the surface of the silicon wafer. Anisotropic etching of the silicon above and below the calorimeter location forms a suspended membrane, and the bismuth (Bi) and titanium (Ti) thermopile are deposited to form the calorimeter. A second thin layer of Su-8 encapsulates the thermopile, ensuring that the nanocalorimeter is isolated electrically and protected from chemical reactions occurring on the platform. Walls complete a Su-8 polymer-lined microfluidic channel for substrate delivery to the reaction zone above the calorimeter (Figure 1B). The nanocalorimeter senses temperature differences using a 27 junction Bi/Ti thermopile (Figure 1C). Sensing and reference junctions are arranged in 500 μm diameter semicircles. The low thermal conductance and thin profile of the membrane minimizes heat flux away from the sensing elements into the silicon base, thermally isolating the reaction zone on the nanocalorimeter to ensure high sensitivity. Both junction sets are located in the microfluidic channel, which allows for compensation for unwanted reaction enthalpies in differential calorimetry. We used this to subtract the heat of dissolution by exposing the reference junctions with denatured enzyme [19].

The thermopile self-generates a voltage difference proportional to a difference in temperature between the sensing and reference junctions. The output voltage is amplified by a 10,000x, custom-built low-noise amplifier, and recorded using a custom National Instruments LabVIEW software module. In order to verify our results from modeling and determine the LOD for TELISA on the nanocalorimeter platform, simplified experiments measuring the amounts of enzyme were performed using CAT and hydrogen peroxide. Experiments were performed on the platform by first depositing a 10 nl volume of enzyme onto the area directly above the nanocalorimeter sensing junctions. Once the small volume was dried, the lid was added to form the microfluidic channel, and the platform was connected to the electrical amplifier. A total of 650 nl of substrate was placed at the entrance to the microfluidic channel and was drawn in by capillary force, filling the entire channel. This filling created a large signal artifact lasting approximately 100 milliseconds, obscuring any signal during that time. The reaction began when the substrate reconstituted the dried enzyme, producing heat in the reaction volume above the sensing junctions. Since the heat-producing reaction occurred in the channel with both the sensing and reference sets of junctions of our differential calorimeter, thermal diffusion modeling was important when designing assays, to ensure that a detectable change in temperature was produced at the sensing junctions before the heat diffused through the channel to the reference junctions.

### 2.2. Model Construction

**Figure 2:**
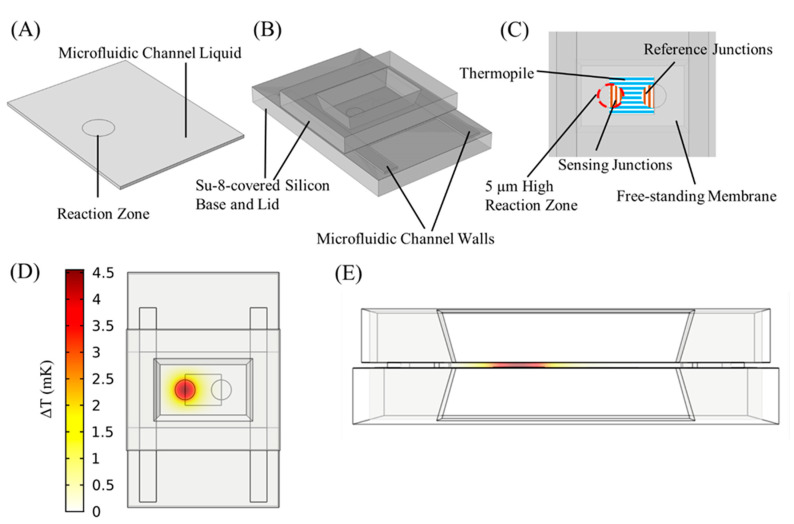
Three-dimensional (3D) calorimeter platform model, constructed in COMSOL Multiphysics. (**A**) The microfluidic channel liquid is represented in the model by a block, designated as water for physical and thermal properties and assigned an initial homogeneous substrate concentration. (**B**) The nanocalorimeter platform, consisting of the base, lid, walls, and membrane containing the calorimeter thermopiles, was added to the microfluidic channel liquid. (**C**) The sensing and reference junctions are simplified to uniform half circles that average the temperature differences between them. The Bi/Ti thermopile tracks between the junctions are modeled in the volume between the two half-circles. Top-down (**D**) and cross-section (**E**) spatial distribution of heat at 0.43 s, at which temperature difference between the reference and sensing junctions was the greatest.

To model TELISA performed on the platform, a three-dimensional (3D) model was constructed in COMSOL Multiphysics. Figure 2A shows the model representation of the sample liquid within the microfluidic channel, consisting of a block 2355 μm wide by 3000 μm long by 50 μm tall. A cylindrical volume of diameter 500 μm and height 5 μm in the channel liquid is designated as the reaction zone, where the enzymatic reaction governed by Michaelis–Menten kinetics occurs. The microfluidic channel liquid and reaction zone were defined as water to properly simulate the diffusion of substrate and enzyme, as well as heat capacity and thermal conductivity to compute temperature profiles. The 3D model was then extended to include the calorimeter platform (Figure 2B) and assigned material-related thermal properties *G_i_*, taken from our previous modeling effort, to simulate the calorimeter platform’s thermal response to the heat input from the enzymatic reaction [19]. The liquid channel was confined by the two thin Su-8 membranes and the two Su-8 channel walls with thermal properties *G_mem_* (thermal conductivity *k* = 0.2 W/(m*K); density *ρ* = 1123 kg/m^3^; heat capacity *C_p_* = 1200 J/(kg*K)), and the sensing thermocouple junctions were embedded within the membrane. The sensing and reference junction areas were simplified as cylinders within the membrane, with effective thermal conductance *G_junct_* (*k* = 0.46 W/(m*K); *ρ* = 1268 kg/m^3^; *C_p_* = 1180 J/(kg*K)), and the thermopile tracks as a rectangular block between them, with effective thermal conductance *G_therm_* (*k* = 2.4 W/(m*K); *ρ* = 2346 kg/m^3^; *C_p_* = 1034 J/(kg*K)), as seen in Figure 2C. This can be done because the thermocouples within the differential thermopile calorimeter add temperature differences, but in a manner that makes a temperature difference at one pair of thermocouples indistinguishable from another in the generated signal. Due to the thermocouples’ close proximities to each other and the high thermal conductivity of the aqueous environment above them, the sensing and reference junctions can each be simplified to uniform regions that average the temperature differences between them in an amplified voltage signal. The effective conductance *G_junct_* and *G_therm_* were determined by the proportional volumes of the membrane, Bi, and Ti within the respective regions. The lid and base were designated as silicon within COMSOL’s included material library and the membrane as Su-8 polymer (*G_mem_*) for the material properties required to simulate heat flow through the platform. The portions of the membrane embedded with the nanocalorimeter were assigned their specific heat and thermal conductivity values for the respective ratios of Su-8, bismuth, and titanium within each volume.

### 2.3. Model Operation and Data Processing

Thermal ELISA reactions were simulated using the COMSOL Transport of a Diluted Species physics suite, which models the concentration field of a dilute solute in a solvent. The microfluidic channel liquid was assigned a homogeneous initial concentration of a substrate. A chemical reaction was assigned within the reaction volume, which reduced the substrate concentration over time as defined by the Michaelis–Menten kinetics of the enzyme to be simulated. CAT was explored. The simulation was run for 30 s, with the reaction beginning at *t* = 0.05 s to offset for the filling noise. In order to find the amount of substrate consumed by the reaction, the substrate was integrated across the microfluidic channel liquid and subtracted from the integral of the previous time point. This change in substrate per Δ*t* was multiplied by the enthalpy of the decomposition of hydrogen peroxide to find the heat produced by the reaction, and assigned as an energy source located within the reaction zone. Using the COMSOL Heat Transfer in Solids suite, the heat transfer through conduction, convection, and radiation was simulated, as shown in Figure 2D. The differential temperature between the sensing and reference junctions was multiplied by the total calorimeter Seebeck coefficient to yield a predicted electrical output signal. The cross-section in Figure 2E demonstrates that the 50 µm high microfluidic channel generates a large heat gradient from the reaction zone across the width of the channel, creating the temperature difference measured between the sensing and reference junctions. Thus, the reference junctions can be located within the same channel for differential calorimetry, instead of being thermally anchored to the silicon substrate. For a TELISA at a given substrate level, the model can be varied over inputs of enzyme amount (*Ea*), enzyme turnover rate (*kcat*), and the inactivation factor (*InF*). The inactivation factor models the inactivation of catalase after (*InF* × 10^7^)^−1^ number of turnover events. The modeled signal *y’* was compared to experimental TELISA signal y by root mean square error (RMSE).

(1) g(Ea,kcat,InF)=y’(n)

(2) t=n∗Δt

(3) $$\mathrm{RMSE}=\sqrt{\frac{\sum_{1}^{n}{\left(y-y^{\prime }\right)}^{2}}{n}}$$

### 2.4. Catalase Experiment

Bovine liver catalase was obtained from Fisher Scientific (2190015MU). All solutions were made using 1× PBS Buffer (Thermo Scientific 28348). Suspended CAT was allowed to dry over the sensing thermocouple junctions of the calorimeter, and the capillary channel was assembled. The channel was then filled with dilute H_2_O_2_, by placing a drop at the entrance of the channel and allowing capillary forces to draw the liquid in. The voltage of the thermopile was recorded over time. After a voltage peak, the signal then formed a baseline dependent on the diffusion of substrate into the reaction zone, the reaction rate of the enzyme, and the active enzyme amount remaining after inactivation from past turnover events. Calorimeter platforms were cleaned between experiments using deionized water and isopropanol.

## 3. Results and Discussion

### 3.1. Enzyme-Based Model Operation

**Figure 3:**
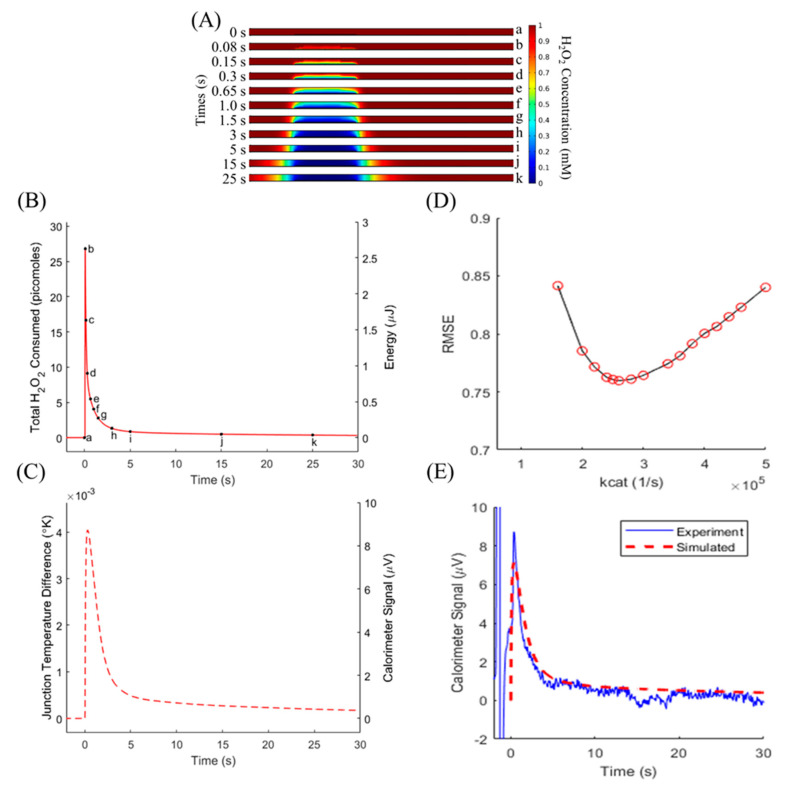
(**A**) Cross-sections across the width of the microfluidic channel show H_2_O_2_ depletion over time. (**B**) Total substrate consumed over time (left axis) was converted to the energy released within the reaction zone (right axis). A quick spike of heat was released (a–b), then quickly decreased as all initial substrate within the reaction zone was consumed (c–h). The substrate slowly diffused into the reaction zone and was quickly consumed, approaching a steady state (i–k). In steady state, there is a finite amount of substrate diffusing into the reaction zone, which results in the signal not returning to baseline. The relatively large concentration and volume of substrate maintains the steady state above baseline. (**C**) Temperature difference between sensing and reference junctions (left axis) and predicted voltage generated by the thermopile during the reaction (right axis). (**D**) Root mean square error (RMSE) minimization found the best fit over the full 30 s of simulation, allowing for the enzyme parameter (*kcat*) determination. (**E**) Model and experiment comparison for 1 mM initial H_2_O_2_ and 10 femtomoles of catalase. Results from the calorimeter response model (red dashed line) closely match experimental (blue line) data.

Diffusion modeling, shown in Figure 3A, shows the progression of substrate concentration within the microfluidic channel over the time course of the simulation. In Figure 3A, beginning with a homogeneous concentration (a), the H_2_O_2_ within the reaction zone was consumed within the first second (b–f). The reaction then sharply slowed as the substrate diffused into the reaction zone (g–k). The change in H_2_O_2_ concentration was converted to the total substrate consumed (Figure 3B, left axis), and multiplied by the reaction enthalpy to find the energy produced by the reaction over time (Figure 3B, right axis). A quick spike of heat was released (a–b), then quickly decreased as all initial substrate within the reaction zone was consumed (c–h). Substrate slowly diffused into the reaction zone and was quickly consumed, approaching a steady state (i–k). The energy curve was assigned as a heat source within the reaction volume, where it produced a change in temperature set by the specific heat of the liquid, which was treated as water due to the dilute nature of the H_2_O_2_ substrate. Heat diffusion through the connected membrane and surrounding liquid was simulated, governed by the assigned thermal conductivities. Figure 3C shows the temperature difference between the sensing and reference junction regions of the membrane (left axis), which was converted to a predicted calorimeter signal by multiplying by the Seebeck coefficient of the thermopile. With the inputs of substrate concentration, enzyme amount, and kcat, the model predicts the full time course of the reaction as measured by the nanocalorimeter platform.

### 3.2. Validation of Numerical Model

The model was first evaluated at a substrate concentration of 1 mM H_2_O_2_ to avoid enzyme inactivation effects. The enzyme amount of 10 fmol CAT quickly consumed the local substrate within the enzyme volume, then was limited by diffusion. In order to minimize the error between the predicted and experimentally-measured signals, the *kcat* value governing the rate of the CAT reaction was iterated over a range of values (Figure 3D). RMSE was minimized at a *kcat* value of 260,000 1/s. The temperature difference between sensing and reference junctions produced a simulated signal that closely followed the experimental signal (Figure 3E). This confirms that the model has improved from previous iterations to include enzyme kinetics, extending its utility from calorimeter sensitivity modeling to include enzyme-based assay design and model-assisted determination of assay results [19].

### 3.3. Model Adaptation at High Substrate Concentration

**Figure 4:**
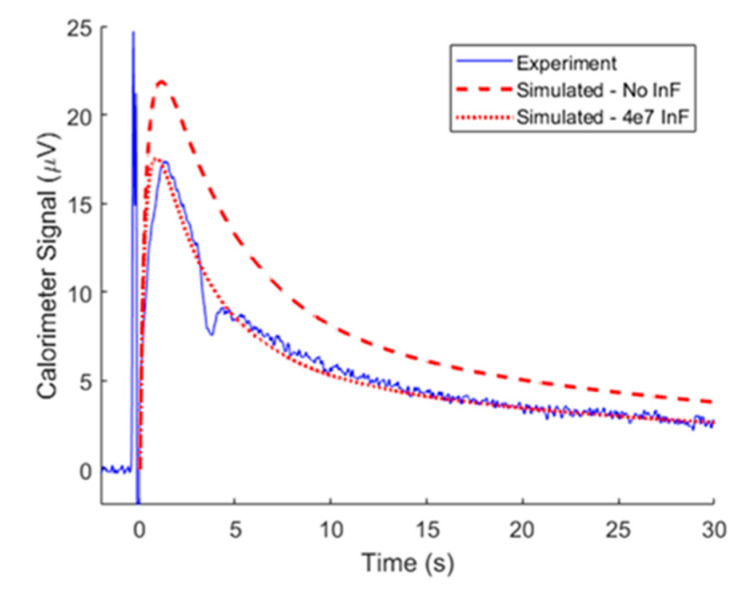
Time course of calorimeter output (blue) at substrate concentration of 10 mM H_2_O_2_ and simulated signals with (red dashed line) and without (red dotted line) enzyme deactivation.

Increased H_2_O_2_ increased the maximum rate of turnover, which produced a greater magnitude signal. This can be seen in the relative magnitudes of the experimental signals (solid blue) in Figure 3E and Figure 4. Figure 3E shows a peak amplitude of 9 µV for 10 femtomoles of catalase and 1 mM concentration of H_2_O_2_, whereas the experiment in Figure 4 peaked at 17 µV for only 2.5 femtomoles of catalase, but 10 mM H_2_O_2_. This amplified signal improved the sensitivity of a TELISA performed on the nanocalorimeter platform. The effect lessens as the substrate concentration approaches the enzyme *K_m_* constant, when the enzyme is saturated with substrate. This sets an upper limit of the *K_m_* value of 93 mM H_2_O_2_ [22]. At substrate concentrations greater than 10 mM, the oxygen gas produced by the reaction formed bubbles within the reaction zone. These bubbles reduced and moved the reaction zone away from the sensing junctions of the calorimeter, and were impossible to model, so initial substrate concentrations were limited to 10 mM. CAT deactivates in the presence of high concentrations of H_2_O_2_ and after approximately 10^7^ turnover events [18,23]. By adding a component that reduced the amount of active enzyme proportionally to the change in substrate concentration (inactivation factor *InF*), model results improved in matching experiments using high H_2_O_2_ concentrations. As seen in Figure 4, TELISA with 10 mM H_2_O_2_ model and experimental results agreed with enzyme parameters of *Km* of 93 mM, *kcat* of 100 × 10^3^ 1/s, and deactivation after 4 × 10^7^ turnover events. We have observed loss of activity in CAT over time, and enzyme degradation is accelerated by a number of factors, so the change in *kcat* from previous runs was expected [24,25].

### 3.4. Model-Assisted TELISA

**Figure 5:**
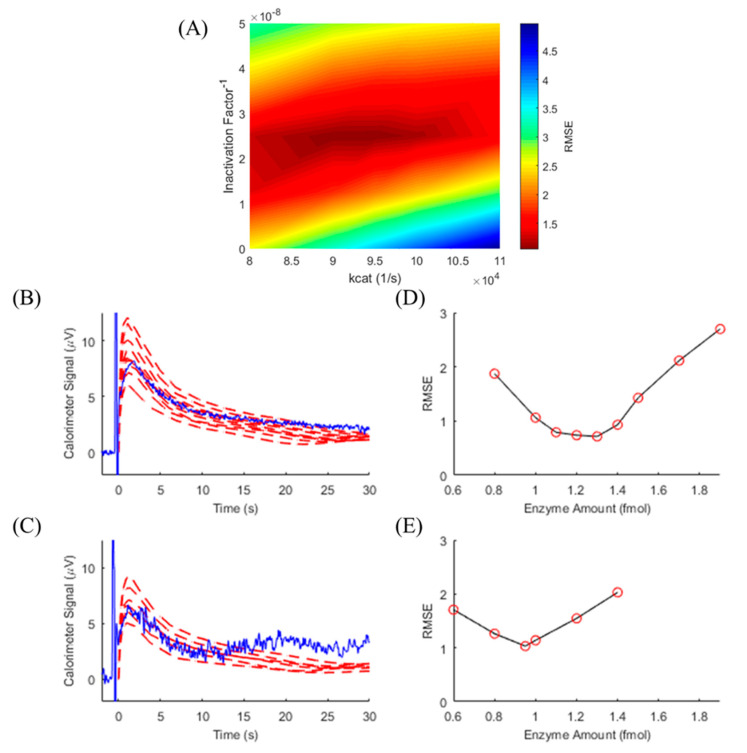
(**A**) RMSE surface for enzyme parameter calibration for thermometric, enzyme-linked immunosorbent assay (TELISA). With 2.5 fmol of catalase (CAT) and 10 mM H_2_O_2_ held constant, enzyme parameters *kcat* and *InF* were varied in the model and compared to 30 s of the experimental signal to determine best fits for the conditions. These parameter values were then used for subsequent modelling to determine unknown enzyme amounts. Calorimeter output (blue) is shown for TELISA with 10 mM H_2_O_2_ at 1.5 fmol (**B**) and 1.0 fmol (**C**) of CAT. Modeled signals (red dashed line) were generated using *kcat* and *InF* values from the calibration step and enzyme amounts shown as a red circle in the corresponding figures (**D**) and (**E**). RMSE minimization from the simulated signals allow for the determination of the enzyme amount.

TELISA relies on knowing the reaction kinetics of the enzyme generating the heat-producing change. These characteristics can be determined by calibrating the reaction with a known enzyme amount. CAT-based TELISA experiments were performed at 10 mM H_2_O_2_ substrate levels on multiple devices and days. In order to calibrate for differences in enzyme and platform performance, TELISA experimental results with the known amount of 2.5 fmol CAT were compared to model results iterated over ranges of *kcat* and *InF* values. RMSE was minimized, as seen in Figure 5A, and model-determined best-fit *kcat* and *InF* values were then used to determine unknown *Ea* values for the subsequent TELISA experiments run on the same device and day. Over four discrete runs (*n* = 4), the minimized model RMSE (Figure 5B–E) returned 1.80 ± 0.54 fmol for the 1.5 fmol experiments and 1.04 ± 0.37 fmol for the 1 fmol experiments. We estimate the primary source in error to be related to experimental factors. The TELISA protocol required repeated deposition of exact volumes of the enzyme, and pipette calibration showed an average error of 6.5%. This, combined with inaccuracies in weighing reagents and differences in placement location of the enzyme in relation to the calorimeter sensing junctions, all contributed to the error seen in the TELISA results.

### 3.5. Determining TELISA Limit of Detection

**Figure 6:**
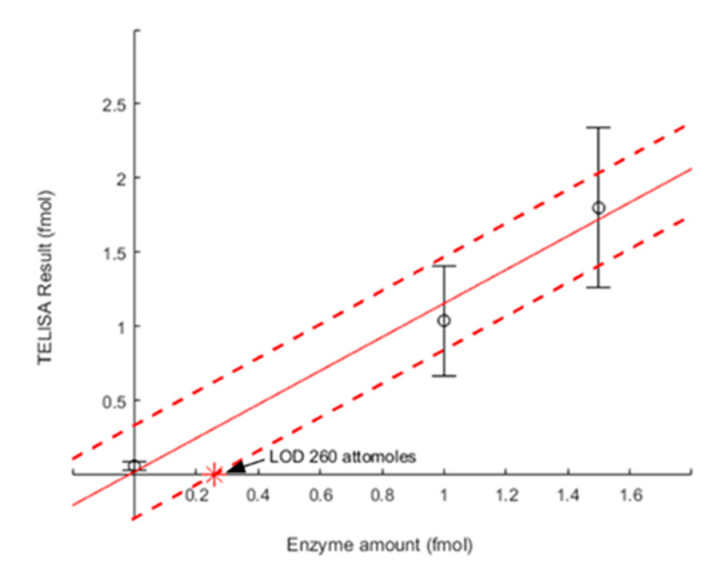
LOD for CAT-based TELISA on a nanocalorimeter platform. Experimental error determined using fixed substrate concentrations of 10 mM H_2_O_2_ and multiple experiments. The LOD of 260 attomoles of CAT was found where the average standard deviation (red dashed line) of the model-assisted determination of the enzyme amount intersects with the *x*-axis.

The extrapolated LOD of the CAT-based TELISA at 10 mM substrate was calculated as the enzyme amount where the average standard deviation (Figure 6, dashed red line) of the model-assisted determination of the enzyme amount intersected with the *x*-axis. At the intersection, the signal generated by the enzyme is dominated by background noise. The extrapolated LOD was found to be 260 attomoles. Table 1 shows the LOD comparison between this approach, previous phenomenological TELISA methods, and a different nanocalorimeter design. This is a considerable improvement over the 25 fmol acid–base neutralization LOD reported earlier [19]. Furthermore, this represents a significant improvement on the 43.8 picomole LOD of the flow-through TELISA assay [17]. The improvement is due to the model-assisted interpretation of a reaction occurring over time and enzyme calibration, instead of phenomenological determination of either the signal peak or decay time constant [19,20]. The calibration step accounted for changes in the enzyme activity at the times of the experiments.

Measurements at 1 fmol of catalase were significantly different than 0 fmol, giving a measured LOD of femtomoles of catalase. Although the LOD was improved, the measured TELISA limit of quantification did not achieve attomole sensitivity. Measurements at 1 fmol catalase were not significantly different than 1.5 fmol. Most of this error is experimental in nature, and unrelated to the device-based model calibration and determination. Error sources include volume and location inconsistencies when depositing the enzyme onto the nanocalorimeter platform. Enzyme diffusion out of the reaction zone was not considered by the model, which would reduce the calorimeter signal over time. Catalase diffuses much slower than H_2_O_2_, due to the relative molecular sizes, but this could have introduced error between the experimental and simulated signals, since we are considering a longer reaction time course. Enzyme diffusion simulation can be added to the model, but future work plans to include enzyme immobilization to the sensing region surface, in order to retain activity within the reaction zone and eliminate signal fall-off [26]. The 10 mM substrate assay can be used from the LOD to a maximum of 3 femtomoles, when the reaction forms oxygen bubbles at the reaction site, affecting the heat signal. The range of the assay can be extended by reducing the substrate concentration, allowing for higher enzyme amounts to be probed. Signal linearity will depend on application-dependent factors, including functionalization effects on the enzyme and matrix effects from the biological sample. By using the model to investigate 30 s of data for enzyme amount determination, assay design was no longer limited to enzyme/substrate ratios that produce a large initial spike of the signal. TELISA results were also adjusted for changing enzyme kinetics and platform conditions by determining enzyme kinetics as model inputs with a calibration experiment of a known amount of enzyme.

## 4. Conclusions

A finite element numerical model was constructed to compute the calorimeter response to an enzyme reaction. The simulated calorimeter signal closely follows experimental results for CAT. Determination of enzyme parameters through calibration is a necessary step to improve the robustness of the modeling and track changing enzyme kinetic characteristics. Model-assisted TELISA improves on previous methods to determine label enzyme amounts on the calorimeter platform. Our high-resolution nanocalorimeter platform, combined with modeling, projects to the attomole limit of detection of a CAT for a TELISA. In order to achieve this LOD, the labeled analyte would need to be delivered directly to the reaction volume without any loss. Future work will focus on validating TELISA in a comprehensive clinical study before developing point-of-care applications for this adaptable platform.

## Figures and Tables

**Table 1:** TELISA limit of detection (LOD) comparison.

Source	TELISA Quantification	LOD (Femtomole)
Flow-injected immunosorbent column (Mecklenburg et al. [11])	Baseline shift	86,000
Flow-injection nanocalorimeter (Xu et al. [17])	Baseline shift	43,800
Capillary nanocalorimeter (Kazura et al. [19])	Phenomenological	25
Model-assisted capillary nanocalorimeter	Model-assisted signal interpretation	0.260
